# A novel anatomical description of the esophagus: the supracarinal mesoesophagus

**DOI:** 10.1007/s00464-023-10109-7

**Published:** 2023-06-14

**Authors:** Miguel A. Cuesta, Romy C. van Jaarsveld, Fernando Mingol, Ronald L. A. W. Bleys, Richard van Hillegersberg, Carmen Padules, Marcos Bruna, Jelle P. Ruurda

**Affiliations:** 1 Department of Surgery, University Medical Centre Amsterdam, Amsterdam, The Netherlands; 2 Department of Surgery, University Medical Centre Utrecht, Heidelberglaan 100, 3584 CX Utrecht, The Netherlands; 3 Department of Surgery, Hospital Universitario La Fé, Valencia, Spain; 4 Department of Anatomy, University Medical Centre Utrecht, Utrecht, The Netherlands; 5 Department of Anatomy, Facultad de Medicina, Universidad Complutense, Madrid, Spain

**Keywords:** Esophagectomy, Prone position, Thoracoscopy, Left recurrent laryngeal nerve, Mesoesophagus

## Abstract

**Background:**

During thoracoscopic esophageal resection, while performing the supracarinal lymphadenectomy along the left recurrent laryngeal nerve (LRLN) from the aortic arch to the thoracic apex, we observed a not previously described bilayered fascia-like structure, serving as prolongation of the already known mesoesophagus.

**Methods:**

We retrospectively evaluated 70 consecutively unedited videos of thoracoscopic interventions on esophageal resections for cancer, in order to determine the validity of this finding and to describe its utility for performing a systematic and more accurate dissection of the LRLN and its adequate lymphadenectomy.

**Results:**

After mobilization of the upper esophagus from the trachea and tilting the esophagus by means of two ribbons, a bilayered fascia was observed between the esophagus and the left subclavian artery in 63 of the 70 patients included in this study. By opening the right layer, the left recurrent nerve became visualized and could be dissected free in its whole trajectory. Vessels and branches of the LRLN were divided between miniclips. Mobilizing the esophagus to the right, the base of this fascia could be found at the left subclavian artery. After dissecting and clipping the thoracic duct, complete lymphadenectomy of 2 and 4L stations could be performed. Mobilizing the esophagus in distal direction, the fascia continued at the level of the aortic arch, where it had to be divided in order to mobilize the esophagus from the left bronchus. Here, a lymphadenectomy of the aorta-pulmonary window lymph nodes (station 8) can be performed. It seems that from there the fascia continued without interruption with the previously described mesoesophagus between the thoracic aorta and the esophagus.

**Conclusions:**

Here we described the concept of the supracarinal mesoesophagus on the left side. Applying the description of the mesoesophagus will create a better understanding of the supracarinal anatomy, leading to a more adequate and reproducible surgery.

Because of the augmentation and perfect visualization obtained during minimally invasive surgery anatomical landmarks of the operative field can be determined more precisely. Since our description of the thoracic mesoesophagus, also known as the aorto-esophageal ligament [1], as observed while performing esophagectomy by thoracoscopy in prone [2], we focused on standardizing the supracarinal anatomy [3]. A difficult part of this intervention is the approach of the left recurrent laryngeal nerve (LRLN) in order to perform an adequate lymphadenectomy of this area without damaging the nerve. There are different ways for this approach [4], but in our experience the looping method, in which the esophagus is tilted by means of two ribbons seems the most ideal technique. Moreover, an extended mediastinal lymphadenectomy is currently advised for every locally advanced esophageal cancer after neoadjuvant therapy [5]. Visser et al., studying the Dutch registered patients (2698 patients) who underwent an esophagectomy after chemoradiotherapy found a clear association between lymph node yield and overall survival, indicating a therapeutic value of extended lymphadenectomy during esophagectomy [6, 7].

It is important is to clarify the surgical anatomy of this area to standardize the surgical approach. In this way, during the thoracoscopic phase of the intervention we have observed a fascia localized in the supracarinal mediastinum, running from the left subclavian artery to the esophagus where the LRLN and lymph nodes are located. As a result of this, a retrospective study of the intraoperative videos of 70 patients, intervened in this way, was performed to define this anatomical concept, important for the approach of the left supracarinal stations and the standardization of this lymphadenectomy.

## Materials and methods

### Retrospective study

All 70 unedited videos were reviewed and evaluated by the two surgical teams (principal surgeons FM and MAC), to assess the constancy, the validity, and the importance of the fascia. This study was carried out in accordance with the applicable legislation reviewal of the Hospital Universitario La Fé in Valencia.

### Video database and surgical team

Complete unedited videos of 70 consecutive Minimally Invasive Esophagectomy (MIE) for cancer from the database of the Upper Gastrointestinal Unit in Hospital Universitario La Fé in Valencia were used. The MIE interventions were performed between 2018 and 2021 by two surgical teams (FM and MAC) in slight thoracoscopic semiprone position with a left cervical anastomosis. Besides the surgical video, the database contains general data and patient characteristics, operative records, perioperative data, the occurrence of peri- and postoperative complications, and the number of removed lymph nodes. Videos and their corresponding data were included after written informed consent. Videos were stripped from any patient or surgeon identifiers.

### Patient characteristics

These involved 47 adenocarcinomas and 23 Squamous cell cancer of patients with an average age of 64 years (45–83 years). All of these were considered advanced but resectable and the patients received as neoadjuvant treatment the chemoradiotherapy according to the CROSS scheme [8]. All interventions were performed as curative intent. The interval between neoadjuvant therapy and intervention was on average eight weeks.

### Surgical procedure

In all cases a total lymphadenectomy of the upper mediastinum was performed through a right thoracoscopy in prone position with a slight inclination in semi prone position of near 10 degrees.

Dissection of the supracarinal esophagus and lymphadenectomy.

**Fig. 1:**
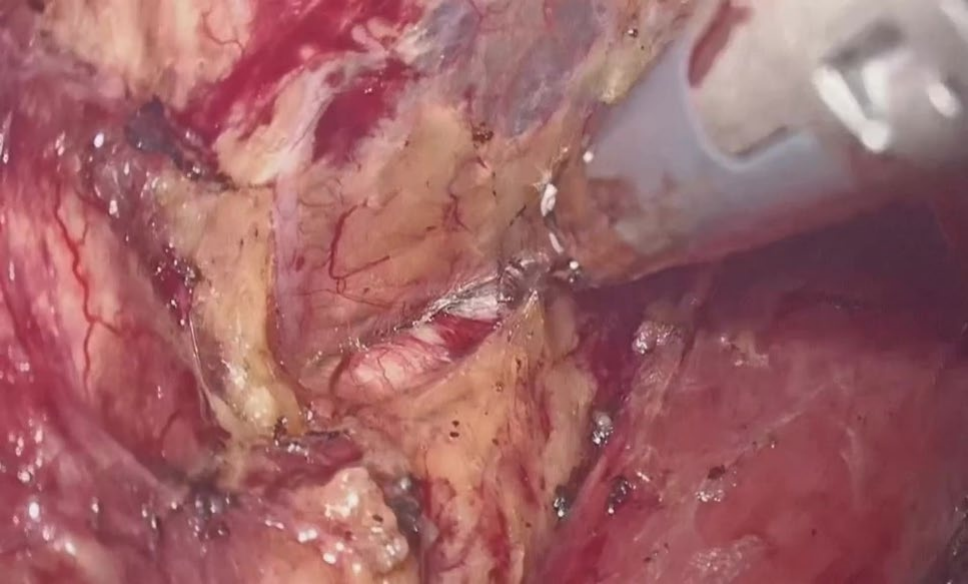
Left subclavian artery, the root of the SML is dissected

**Fig. 2:**
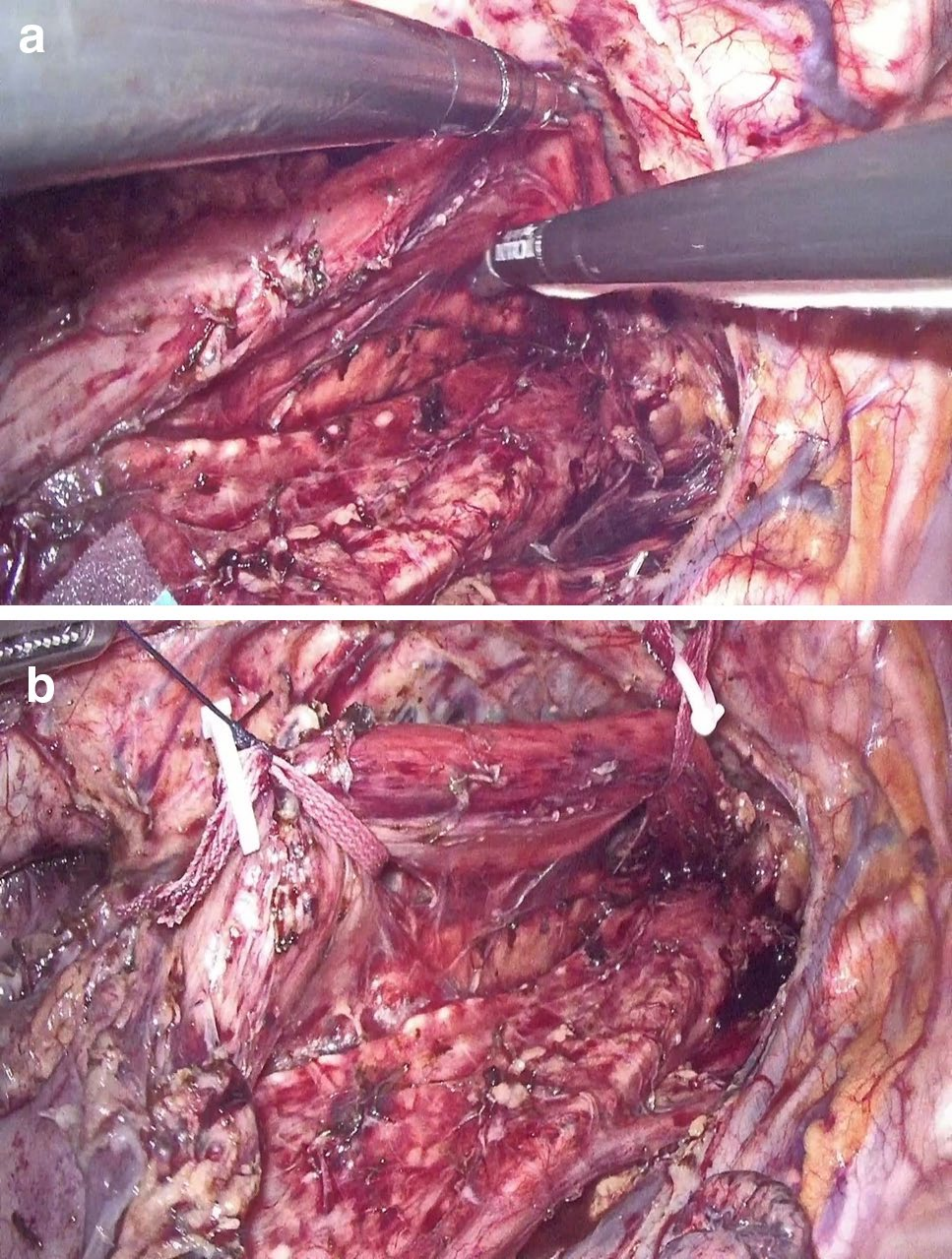
**a**, **b** SML is visible as a curtain after traction of the upper esophagus

**Fig. 3:**
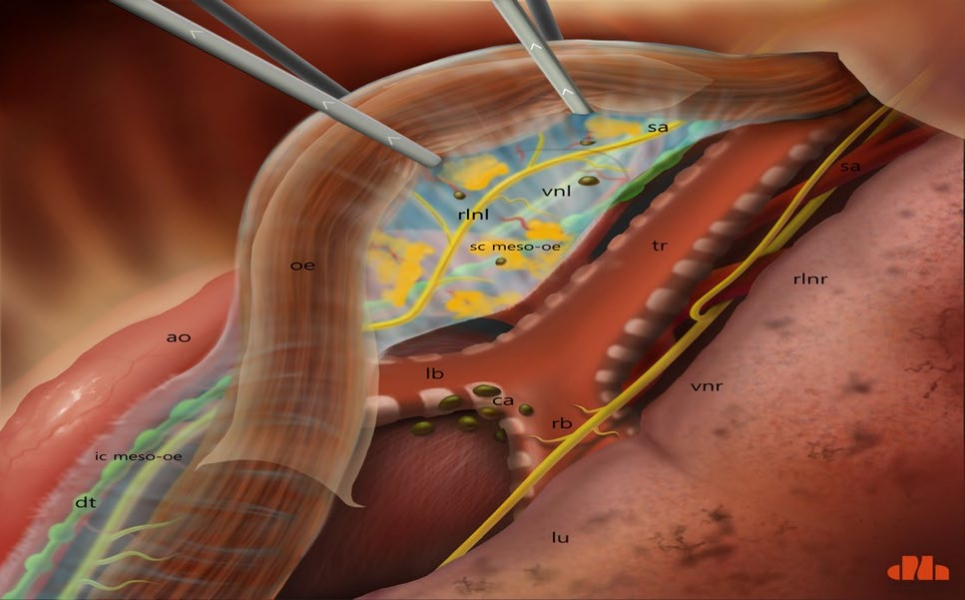
Schematic presentation of a dorsoventral view of the SML, from right thoracic cavity. ao: aorta; dt: thoracic duct; ic meso-oe: infracarinal mesoesophagus; oe: oesofagus; rlnl: left recurrent laryngeal nerve; ca: carina; lb: left bronchus; rb: right bronchus; tr: trachea; vnl: vagal nerve left; sc meso-oe: supracarinal mesoesophagus; sa: left subclavian artery; vnr: right vagal nerve; rlnr: right recurrent laryngeal nerve; lu: right lung

**Fig. 4:**
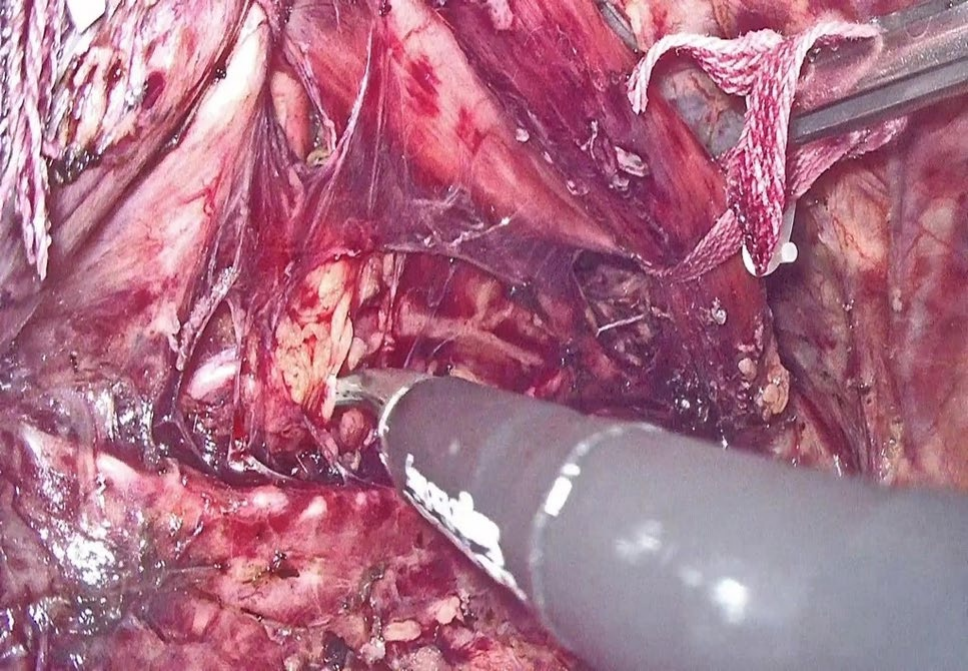
Under traction of the esophagus by two ribbons, the medial layer is opened, the LRLN is found, and lymphadenectomy can be started

**Fig. 5:**
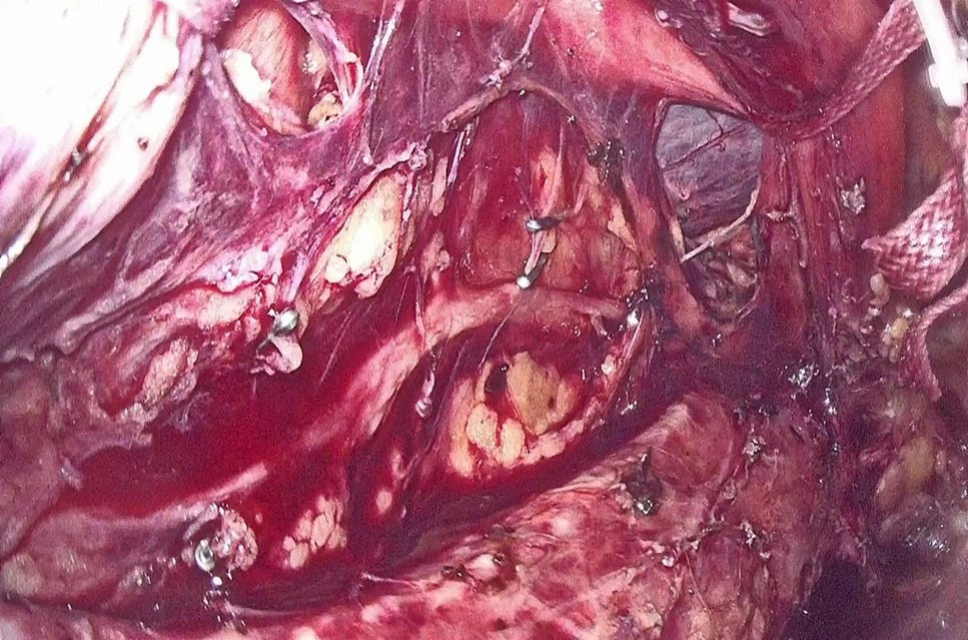
LRLN dissection with division of branches by means of miniclips

**Fig. 6:**
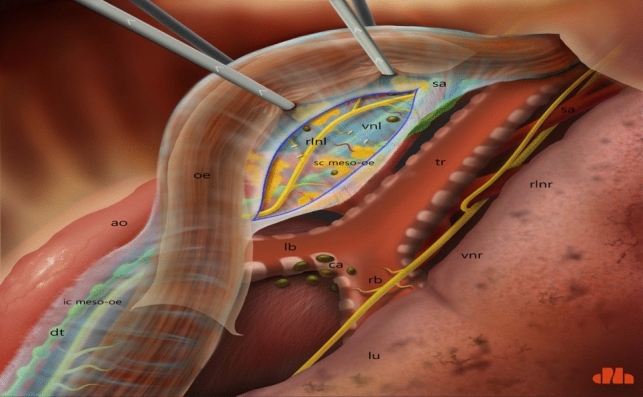
Schematic presentation of the open SML and the dissection of the LRLN and lymphadenectomy of stations 2 and 4L (abbreviations are the same as in Fig. 3)

**Fig. 7:**
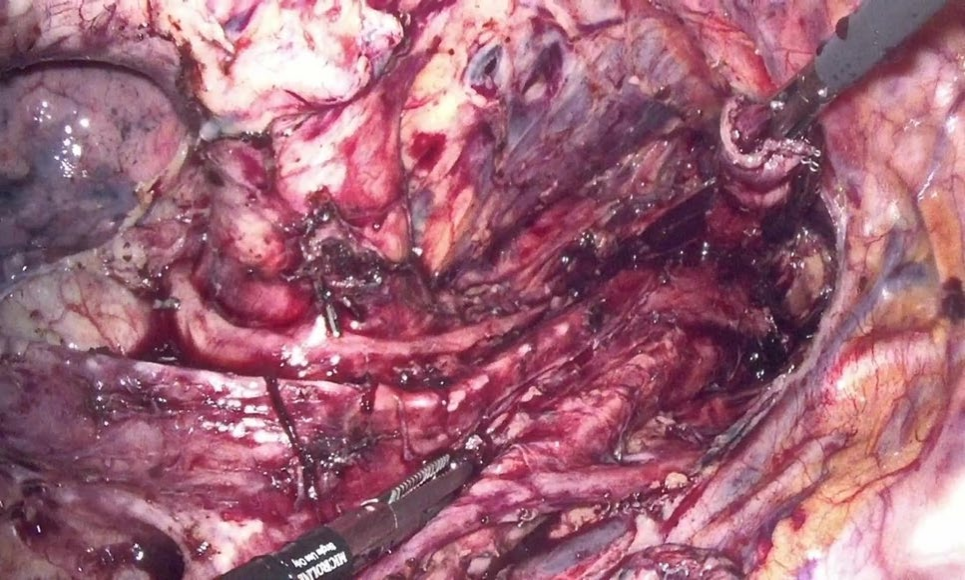
The fascia of the SML continues along the aortic arch

**Fig. 8:**
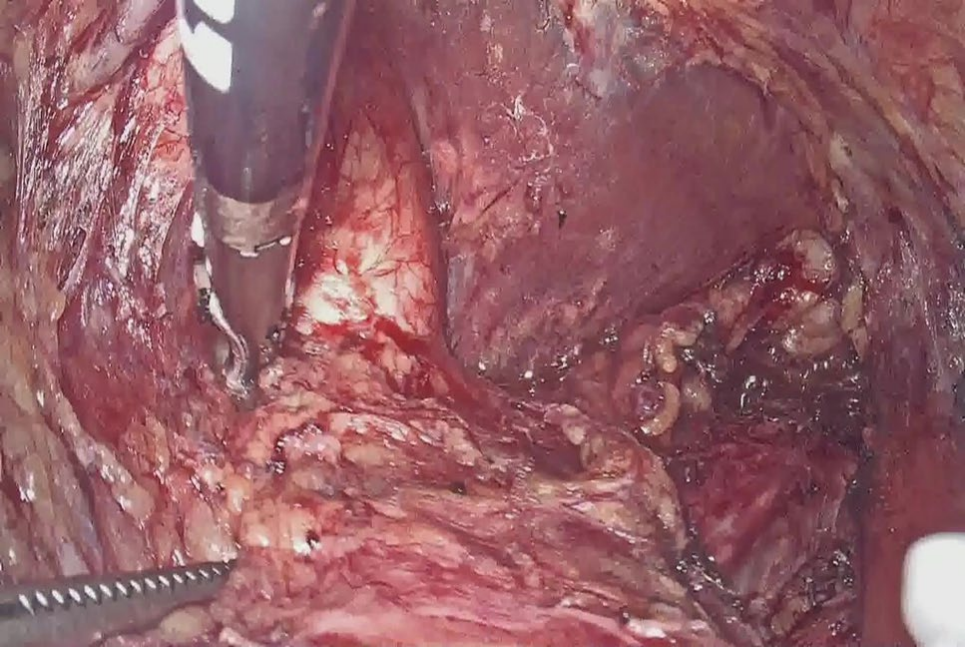
The LRLN dissected at whole length from its recurrence at the aortic arch

**Fig. 9:**
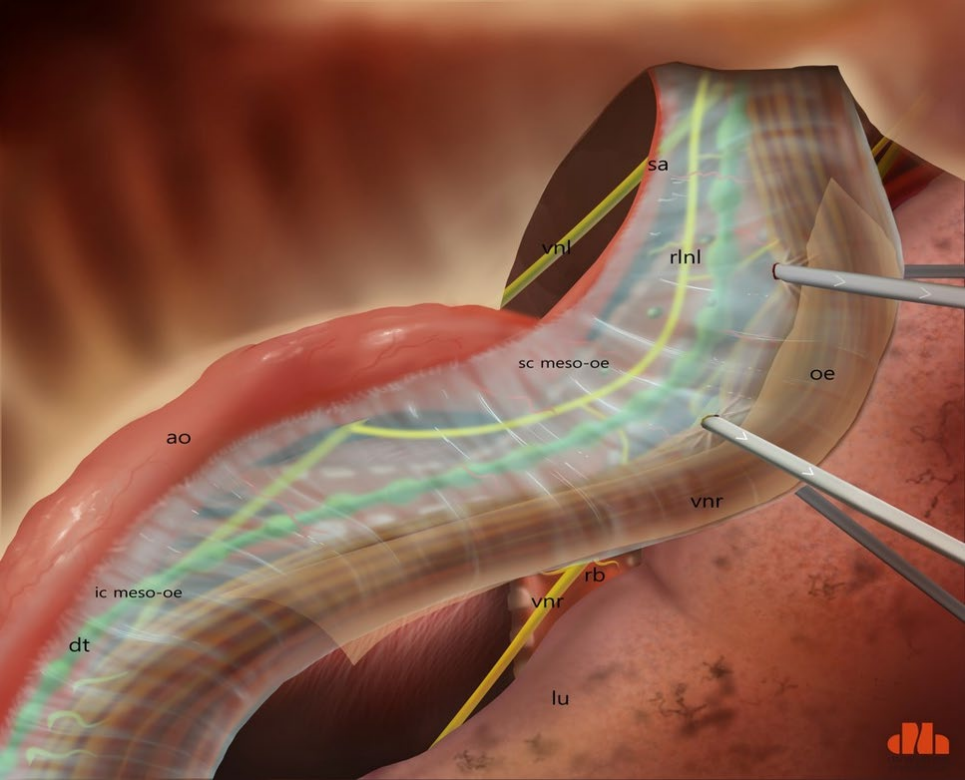
Schematic presentation of the whole concept: prolongation of the SML with the thoracic mesoesophagus. ao: aorta; dt: thoracic duct; lu: lung; rlnl: left recurrent laryngeal nerve; oe: oesophagus; sc meso-oe: supracarinal meso-pesophagus; ic meso-oe: infracarinal meso-oesophagus

1. After lymphadenectomy of the stations 2 and 4R, the esophagus is dissected free from the trachea. The left tracheal groove is dissected as much as possible. After dissection along the left pleura, the left subclavian artery is visualized (Fig. 1).
2. The supracarinal esophagus is tilted by means of two ribbons (passed to the outside). In this way the left supracarinal mesoesophagus (SML) is visible (Fig. 2a and b), from the thoracic apex to the aortic arch. Schematic representation (Fig. 3).
3. The medial layer of the SML is opened and the LRLN, located between the two layers of the fascia is carefully dissected to perform the lymphadenectomy (Fig. 4).
4. Small branches of nerves and vessels are taken down between microclips and lymphadenectomy of the SML is performed (stations 2 and 4L) up to the left subclavian artery (Fig. 5 and schematic representation in Fig. 6).
5. Retracting the esophagus by a ribbon to the right, dissection continues in direction to the aortic arch, where over the whole length the LRLN nerve, recurring at the aortic arch, is dissected free (Fig. 7).
6. The fascia continues without interruption, through the aortic arch to the aorta pulmonary window (Fig. 8) and from there to the descending thoracic aorta (already described as classical thoracic mesoesophagus, schematic representation, Fig. 9).

## Results

The patient characteristics as utilized in the retrospective study are depicted in Table 1. From all patients in the study, 67% had adenocarcinoma and 33% a squamous cell carcinoma.

**Table 1:** Patient characteristics

	Patients (*n* = 70)
Histology	
Adenocarcinoma	47 (67%)
Squamous cell cancer	23 (33%)
pTNM^a^	
Adenocarcinoma	
Stage 0	9 (13%)
Stage I	16 (23%)
Stage II	12 (17%)
Stage III	10 (14%)
SCC^b^	
Stage 0	9 (13%)
Stage I	5 (7%)
Stage II	5 (7%)
Stage III	4 (6%)
Visualization LRLN^c^	
Complete	63 (90%)
Difficult esophageal anatomy	7 (10%)
Average lymph nodes retrieved	
2 and 4L	3
2 and 4R	6
Recurrent nerve LRLN palsy	
Temporary, mostly recovered in 4 weeks	5 (7%)
Definitive	1 (1%)

^a^pTNM: classification for malignant tumors by staging tumor size, lymph node involvement and metastasis

^b^SCC: squamous cell cancer

^c^LRLN: left recurrent laryngeal nerve

In 63 of the 70 patients, after tilting the esophagus by means of two ribbons, a bilayered fascia was found, from the esophagus to the left subclavian artery, and from the upper thoracic aperture to the aortic arch covering the left bronchus. After opening the right layer, the left recurrent nerve was easily found, permitting a complete lymphadenectomy (2 and 4L) with care of the nerve.

The fascia is bilayered and its roots are found at the level of the left subclavian artery. At this level, outside the fascia, the thoracic duct is found.

In seven patients the upper esophagus was markedly displaced to the left of the spine and the looping method could not be performed. In all those seven patients the proximal esophagus was correspondingly divided at the upper part and an adequate upper mediastinal lymphadenectomy was performed.

## Discussion

Since our description of the thoracic mesoesophagus [1], the next project was to standardize the supracarinal anatomy performed by thoracoscopy in prone [2]. The difficult part of this intervention entails the approach of the left recurrent laryngeal nerve to perform an adequate lymphadenectomy of this area without any damage of the nerve.

Having a comprehensive concept of the live surgical anatomy is necessary for ensuring anatomical accuracy as well as for reproducing radical surgical resection for cancer. In the case of esophageal cancer, an extended mediastinal lymphadenectomy is increasingly considered paramount for obtaining a better curation and staging the cancer accurately to improve the prognosis of the patient [5, 7]. Supracarinal lymphadenectomy on both sides is considered by many surgeons to be an essential part of the esophageal resection, independently of the neoadjuvant therapy, but concurrently it implies difficulties because of the possible damage to the recurrent nerves. Although from the theoretically point of view it would be strongly recommended, yet dissection can cause palsy of the recurrent nerves, creating a major influence on the quality of life of the intervened patients. Therefore, a good standardized surgical procedure with an exact knowledge of the surgical anatomy and an adequate step-by-step description of the procedure will be very important. A systematic approach as based on a correct anatomical concept can standardize the procedure, obtaining a good lymphadenectomy with preservation of the recurrent nerves. Our method here described, tries to fix the concept in a correct way, as observed during dissection of the left supracarinal area. In a different way, Fujiwara et al., have defined its supracarinal concept model based on embryological development of the mediastinum and distinguished three layers: a visceral concentric, a vascular, and an outer part. Distinction of these three layers could be of help for an adequate dissection of the supracarinal area [9].

Furthermore, there are different ways to approach the left laryngeal nerve and among them the looping method is the most standardized method [4].

Using this method, the fascia as found by us, and named as supracarinal mesoesophagus, is a constantly occurring anatomical finding. Probably because its base is in the left subclavian artery, we surmise it is a prolongation of the alar fascia. At the cervical level the alar fascia evolved the carotid arteries, jugular veins and vagal nerves on both sides and ended at the posterior middle line of the cervical esophagus. In most of the studies, authors described, at cervical level, the recurrent laryngeal nerves outside this fascia [1, 10]. On the contrary, in our live study, at least the left nerve is localized in the described fascia and not outside it. Therefore, the here described fascia would not be a prolongation of the alar fascia.

Doing the esophagectomy by RAMIE (robot assisted minimally invasive esophagectomy), usually the 4th arm is used to tilt the upper esophagus and, in this way, the LRLN is found, and lymphadenectomy is performed. The facility created by the robot fourth arm makes it less necessary to tilt the esophagus under tension as required during the thoracoscopy in prone position [11]. Therefore, visualization of the described fascia will be reproducible with the two different approaches, MIE (minimally invasive esophagectomy) and RAMIE.

Moreover, an important question is whether a similar supracarinal mesoesophagus will exist on the right side. It is plausible that this supracarinal mesoesophagus does exist on both sides. Nowadays, our thoracoscopic interventions usually start on the right side, followed by dissection of the esophagus and finalizing with the left side. To demonstrate this mesoesophagus on the right side precludes tilting the esophagus before the right side has been dissected. The transmediastinal esophagectomy, in which the esophagus is dissected from the cervical area in the middle may help to define this concept [12]. Currently, to clear the anatomy on the right side, radiological models or cadaver studies are necessary.

Importantly, the extant knowledge of the mediastinal anatomy is based on what we know about its embryological development [13, 14].

## Conclusions

In this article we described the concept of the supracarinal mesoesophagus on the left side of the upper mediastinum. It is a bilayered fascia in which the left recurrent nerve is located, permitting a gentle dissection and complete lymphadenectomy of these stations. Moreover, this fascia continues at the level of the aortic arch and connects with the already described thoracic mesoesophagus.

Applying the description of the supracarinal mesoesophagus will create a better understanding of the supracarinal anatomy, for standardizing the operative technique of adequate lymphadenectomy with preservation of the recurrent laryngeal nerves.
